# Learning to see after early and extended blindness: A scoping review

**DOI:** 10.3389/fpsyg.2022.954328

**Published:** 2022-10-27

**Authors:** Eloise May, Proscovia Arach, Elizabeth Kishiki, Robert Geneau, Goro Maehara, Mahadeo Sukhai, Lisa M. Hamm

**Affiliations:** ^1^School of Optometry and Vision Science, University of Auckland, Auckland, New Zealand; ^2^Benedictine Eye Hospital, Tororo, Uganda; ^3^Kilimanjaro Centre for Community Ophthalmology, Moshi, Tanzania; ^4^Division of Ophthalmology, University of Cape Town, Cape Town, South Africa; ^5^Department of Human Sciences, Kanagawa University, Yokohama, Japan; ^6^Accessibility, Research and International Affairs, Canadian National Institute for the Blind, Toronto, ON, Canada; ^7^Department of Ophthalmology, Faculty of Health Sciences, School of Medicine, Queen's University, Kingston, ON, Canada

**Keywords:** early blindness, deprivation amblyopia, childhood cataract, access to health, neurorehabilitation

## Abstract

**Purpose:**

If an individual has been blind since birth due to a treatable eye condition, ocular treatment is urgent. Even a brief period of visual deprivation can alter the development of the visual system. The goal of our structured scoping review was to understand how we might better support children with delayed access to ocular treatment for blinding conditions.

**Method:**

We searched MEDLINE, Embase and Global Health for peer-reviewed publications that described the impact of early (within the first year) and extended (lasting at least 2 years) bilateral visual deprivation.

**Results:**

Of 551 reports independently screened by two authors, 42 studies met our inclusion criteria. Synthesizing extracted data revealed several trends. The data suggests persistent deficits in visual acuity, contrast sensitivity, global motion, and visual-motor integration, and suspected concerns for understanding complex objects and faces. There is evidence for resilience in color perception, understanding of simple shapes, discriminating between a face and non-face, and the perception of biological motion. There is currently insufficient data about specific (re)habilitation strategies to update low vision services, but there are several insights to guide future research in this domain.

**Conclusion:**

This summary will help guide the research and services provision to help children learn to see after early and extended blindness.

## Introduction

Avoidable visual impairment occurs when someone is unable to access needed and available ocular treatment (most commonly glasses Naidoo et al., 2016), often due to structural inequities (Ulldemolins et al., 2012). Avoidable *blindness* represents the more severe end of this spectrum. A common cause of avoidable blindness is dense, central, bilateral cataracts (henceforth, cataracts). Although common and easily treatable in adulthood, when cataracts are present at birth, outcomes are considerably worse (Bronsard et al., 2018; Allen, 2020). Animal studies in the 1960's famously established that early visual input is required for refined development of the striate visual cortex (Wiesel and Hubel, 1965). This ignited a field of study on plasticity in the visual system (Daw, 2009), including the degree to which auditory and somatosensory cortical representation changes in the absence of visual input (Rauschecker and Harris, 1983; Rauschecker and Korte, 1993). For children with congenital cataracts, prompt ocular treatment is critical (Gogate et al., 2010)–even short delays in treatment mean a child will not develop the same functional vision as their typically developing peers (Lewis and Maurer, 2005, 2009; Maurer et al., 2005, 2007; Birch et al., 2009; Maurer, 2017; Allen, 2020). This secondary visual impairment is sometimes called bilateral deprivation amblyopia, the most severe form of amblyopia in terms of its impact on higher-level perception (Maurer, 2017).

Given the impact, avoidable childhood blindness was a key goal of VISION2020 (Gilbert and Foster, 2001) and is a critical component of the United Nations' Sustainable Development Goals (Sharma, 2021). There are several organizations working toward these goals by targeting prompt treatment of avoidable childhood blindness (Sinha, 2016). For example, the Kilimanjaro Center for Community Ophthalmology (KCCO) provides direct services (including screening, diagnosis, treatment, and low vision for children with avoidable visual impairment and blindness throughout sub-Saharan Africa), trains staff and conducts research on social determinants of eye care (Courtright et al., 2008; Kishiki et al., 2009, 2012, 2016; Kishiki and Courtright, 2012; Reddy et al., 2018). Understanding the visual abilities of newly sighted children influences expectations for results of ocular treatment, as well as assessment and (re)habilitation strategies used to facilitate optimal long-term outcomes. If existing research about bilateral deprivation amblyopia sheds light on the impact of visual deprivation and the possibility to promote development of higher-level visual skills, lessons from this field could be incorporated into ongoing services.

There is some evidence that low vision services beyond magnification, contrast enhancement and classroom positioning can promote visual development even after delays in ocular treatment. Some crucial work in this field stems from early studies linking seminal animal work about plasticity after sensory deprivation to the potential for behavior training in humans (Rauschecker, 1995, 1999). There is a field of study looking at perceptual learning and active training of low-level visual skills (e.g., visual acuity (VA) and stereoacuity) for less severe forms of amblyopia (Rodán et al., 2022), and a few studies addressing (re)habilitation of higher-level visual processing after bilateral deprivation amblyopia (Jeon et al., 2012; Hamm et al., 2018). There is also work on adult recovery after sight restoring visual prosthetics (Beyeler et al., 2017) and low vision (re)habilitation services for children with neurodevelopmental visual processing challenges (Jayaraman et al., 2021; McDowell, 2021), such as cerebral visual impairment “CVI,” and autism. Translating this work to a new clinical population requires dedicated research about visual deprivation, both in terms of measurement of higher-level visual abilities and (re)habilitation strategies. Like Rauschecker (1999), Merabet and Pascual-Leone (2010) remind us of the importance of linking our (re)habilitation strategies to the long history of science about brain plasticity after sensory loss.

What is meant by ‘higher’ or ‘lower’-level perception can be nuanced. There is general consensus that the early stage of visual processing (or lower-level perception) includes the extraction of basic visual features (such as local edges, more global contours, and color) from two-dimensional retinal image. This image-based processing stage is followed by the surface-based processing stage that includes reconstruction of three-dimensional surfaces and estimation of tilt and slant (Palmer, 1999). As visual information moves outside the occipital cortex, perception becomes increasingly complex. For practical purposes (e.g., applications in community settings), higher-level processing has been thought of as occurring in parallel streams, through the adjacent parietal and temporal lobes (Goodale and Milner, 1992). Classically, the ventral stream (within the temporal lobe) is thought to be associated with vision for “recognition” or perception, while the dorsal stream (within the parietal lobe) is thought to be associated with vision for “spatial awareness” or action (Goodale and Milner, 1992; Braddick and Atkinson, 2011; Goodale, 2011). The more recently proposed lateral stream (superior aspect of the temporal lobe) has been suggested to be associated with vision for “social” meaning (Grossmann, 2021; Pitcher, 2021; Pitcher and Ungerleider, 2021; Weiner and Gomez, 2021).

Although such parallel stream hypotheses are widely used, limits of these simplifications are well accepted (de Haan and Cowey, 2011; Freud et al., 2016). One component of complexity is bi-directionality. Within the ventral stream, for example, “top-down” object representations and initial expectations interact with “bottom-up” processing of basic visual features to facilitate recognition (Ullman, 1995; Bar, 2003). Another component of complexity is interaction with non-visual systems. For example, the visual streams overlap considerably with those involved in auditory and language processing (Rauschecker and Scott, 2009).

In terms of challenges faced after bilateral visual deprivation, there is strong evidence that even very short periods of deprivation after congenital blindness has wide implications (Lewis and Maurer, 2005, 2009; Maurer et al., 2005, 2007). Issues within low-level (Lewis et al., 1995), social (Geldart et al., 2002; Le Grand et al., 2004) and spatial (Ellemberg et al., 2002) components of visual processing have been reported. Blindness initiating after the first year of life, but lasting several decades appears to disproportionately impact recognition, and results in profound impairments in interpreting social visual input like faces (Fine et al., 2003; Šikl et al., 2013; Huber et al., 2015). We are interested in children with congenital blindness who have experienced at least 2 years of visual deprivation–the kinds of children who might be seen at a rural screening programme run by organizations like KCCO.

Systematic analyses of literature has focused on understanding critical periods, and mechanisms of visual neurodevelopment (Röder et al., 2021). Our goal was complementary. We wanted to know whether published literature about sight recovery could inform low vision (re)habilitation programs for children after delayed access to ocular treatment. This required inclusion of several disciplinary perspectives, with a variety of methodological approaches, well-suited to a structured scoping review (Peters et al., 2015, 2021; Munn et al., 2018). Our aim was to map literature about the impact of early and extended bilateral visual deprivation, in order to explore the potential utility for updating low vision services for impacted children. We approached this overarching aim with the following four questions:

1. What are the characteristics of the published literature and included participants?
2. How are visual abilities measured and categorized?
3. Which visual abilities are likely to be compromised by (and which are likely to be resilient to) early and extended bilateral visual deprivation?
4. Through what disciplinary lens was the research undertaken and interpreted?

## Methods

### Transparency and openness

This study is reported according to the PRISMA-ScR guidelines (Tricco et al., 2018). We did not pre-register a protocol. Data and will be made available upon reasonable request. This review emerged from a collaborative process including patient, clinical, rehabilitative, eye care systems and vision science perspectives (all contributing authors).

### Definitions and eligibility criteria

We established eligibility criteria according to PICOS [population/problem, intervention/exposure, comparison, outcome, study design (McGowan et al., 2016)], described below.

In terms of population/exposure, we are interested in people who have experienced early (within the first year) and extended (more than 2 years) bilateral visual deprivation, due to a blinding (we defined blindness as a visual acuity [VA] of “counting fingers” or poorer, ~1.5 logMAR) but treatable primary ocular condition (most often dense, central, bilateral cataracts). We use “sight recovery” as a shorthand for the experience of the end of a period of extended congenital bilateral visual deprivation, after ocular treatment has been provided. However, it is important to note that most “sight recovery” patients have residual ocular challenges (nystagmus, strabismus, reduced accommodation, complex refractive needs and secondary complications), and are unlikely to have good VA – “ocular treatment” does not mean “ocular health.” For inclusion, studies needed at least one participant who had experienced sight recovery. We placed no restrictions on age of participants at the time of the study, or duration between ocular treatment and measurement of visual abilities.

Studies with or without a comparison group were included. In terms of outcome, studies needed to include at least one measurement of visual ability beyond standard clinical assessment, reported in a way that sheds light on this unique population. Studies that examined only residual ocular issues, only used standardized clinical measures (e.g., VA), or only reported modeling data or physiological responses, were excluded. We included all study designs and publication dates, including very old manuscripts. Books, reviews, commentaries, editorials, and conference abstracts were excluded. We did not set language limits, rather we sought expertise as needed to interpret non-English publications.

### Information sources and selection of sources of evidence

We searched MEDLINE, Embase and Global Health databases on 23/06/2021 and imported all into Covidence (www.covidence.org). For each included paper we further scanned each references and citing paper for potentially relevant resources and imported these into Covidence iteratively (ending in December, 2021). Duplicates were identified within Covidence, through a combination of automated and manual processes, each manually checked prior to removal. We scanned websites of key author groups for additional information. Within Covidence, two authors independently assessed eligibility in both the title/ abstract, and full text screening stages. Conflicts were resolved by discussion.

### Data charting process

A data extraction form was developed and piloted within Covidence. We started extracting based on the “Data items” listed below. Iteratively, we coded qualitative data items. As part of our “Data synthesis,” emergent codes were turned into new categorical data extraction items. Data extraction was carried out independently by two authors, and consensus achieved by iterative review and discussion. Studies only available in Japanese were screened and extracted by a single author, in discussion with the team.

### Data items and synthesis strategy

#### Question 1: What are the characteristics of the published literature and included participants?

##### Data items

We recorded publication year, country in which study was carried out (where participants were based), and country of the institute within which each author was affiliated. We recorded demographics and clinical characteristics of the participants who met our inclusion criteria, including gender, duration of visual deprivation, time between ocular treatment and measurement of visual ability, as well as VA before and after ocular treatment (if multiple timepoints, we used the last post-treatment time point reported). If there was more than one experiment presented in one study, we only included participant data from the first relevant experiment.

##### Data synthesis

We used a network analysis to visualize co-authorship. Reported VA results were converted to logMAR (where lower values indicate better VA, and 0.0 logMAR is 6/6 in Snellen notation).

#### Question 2: How are visual abilities measured and categorized?

##### Data items

We recorded the type of data collected (simplified to behavioral, impressions and physiology), general approach (quantitative and qualitative) and, if applicable, comparison type (to a reference group and/or across time). We included free text data entry to describe how authors categorized visual skills, and specifics about the visual abilities discussed.

##### Data synthesis

As we extracted data, we coded authors' approaches to categorizing visual skills. Iteratively, we converged on three categories, and went to back through each study to categorize studies broadly as:

1. Hierarchical (e.g., “low” or “mid”-level processing, or parallel hypotheses);
2. Multi-sensory (e.g., visual-auditory or visual-motor integration); and,
3. Functional (e.g., ability to accomplish day-to-day tasks).

Similarly, to generate a consistent framing of visual abilities across studies, we underwent iterative rounds of teasing apart and compiling related visual skills, and simplified them according to approach 1, focusing on the parallel stream hypothesis including a social stream (Grossmann, 2021; Pitcher, 2021; Pitcher and Ungerleider, 2021; Weiner and Gomez, 2021):

1. Primary (e.g., spatial and temporal contrast sensitivity and color perception);
2. Recognition (e.g., identifying shapes and objects);
3. Understanding of visual space (e.g., motion and visual-motor integration; and,
4. Social aspects of vision (e.g., biological motion and face identification).

Classification challenges are explored in the results. We used consistent categories for visual skills in Questions 2 and 3.

#### Question 3: Which visual abilities are likely to be compromised by (and which are likely to be resilient to) early and extended bilateral visual deprivation?

##### Data items

For up to three visual skills per study, we recorded whether authors considered the performance of sight recovery participants within or outside the range of typical performance.

##### Data synthesis

For studies with a reference group, we synthesized reported outcomes for each visual skill. For studies without a reference group, we integrated observations across studies. We focused on outcomes beyond the first 6 months of ocular treatment.

#### Question 4: Through what disciplinary lens was the research undertaken and interpreted?

##### Data items

We allowed free text entry to summarize key insights.

##### Data synthesis

We simplified key insights into categories to shed light on disciplinary lenses. Iteratively, we converged on four categories and went back through each study to note the insights within the following categories:

Quality of life;
(re)habilitation;
Neurodevelopmental insights; and/or,
Impact of timing of ocular treatment on visual skills.

We used a network analysis to guide our description of connections between (non-mutually exclusive) categorical insights, as well as study characteristics (Question 1) and methodological approaches (from Question 2). We also synthesized free text discussion points into a brief narrative summary.

## Results

### Summary of sources of evidence

**Figure 1:**
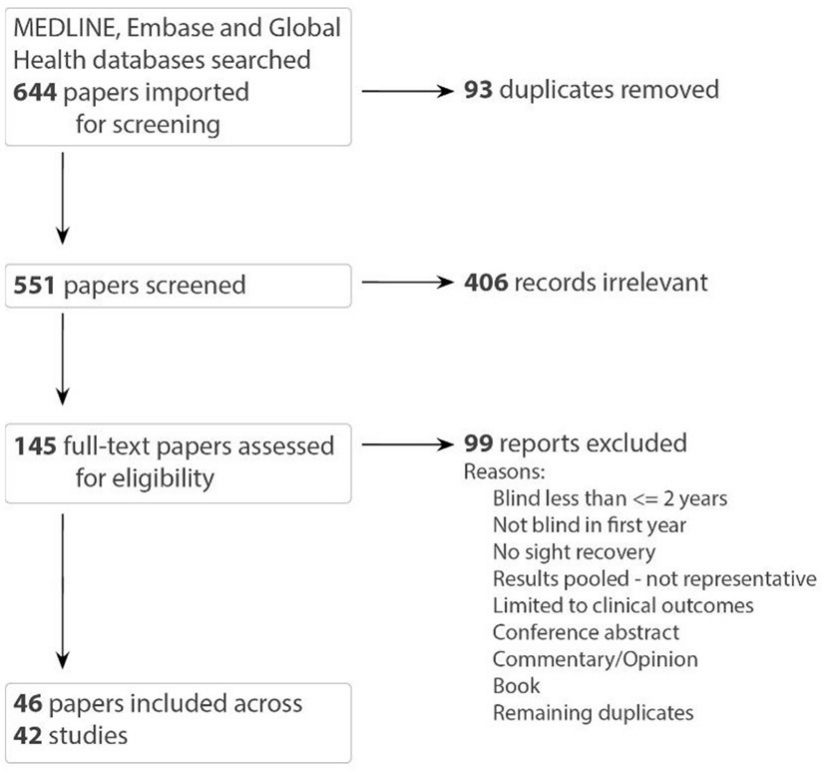
PRISMA flow diagram [papers linked by study: (Umezu, 1987; Umezu et al., 1990, 1991) and (Mochizuki, 1976, 1977, 1979)].

Our search resulted in 644 records. After duplicates were removed, 551 were screened by title and abstract, and 145 were included in the full text screening. Eligible references included 46 papers describing 42 studies, which we reported by study (Figure 1). Papers linked by study include those by Mochizuki (1976, 1977, 1979), Umezu (1987), Umezu et al. (1990, 1991).

#### Question 1: What are the characteristics of the published literature and included participants?

##### Characteristics of the published literature

**Figure 2:**
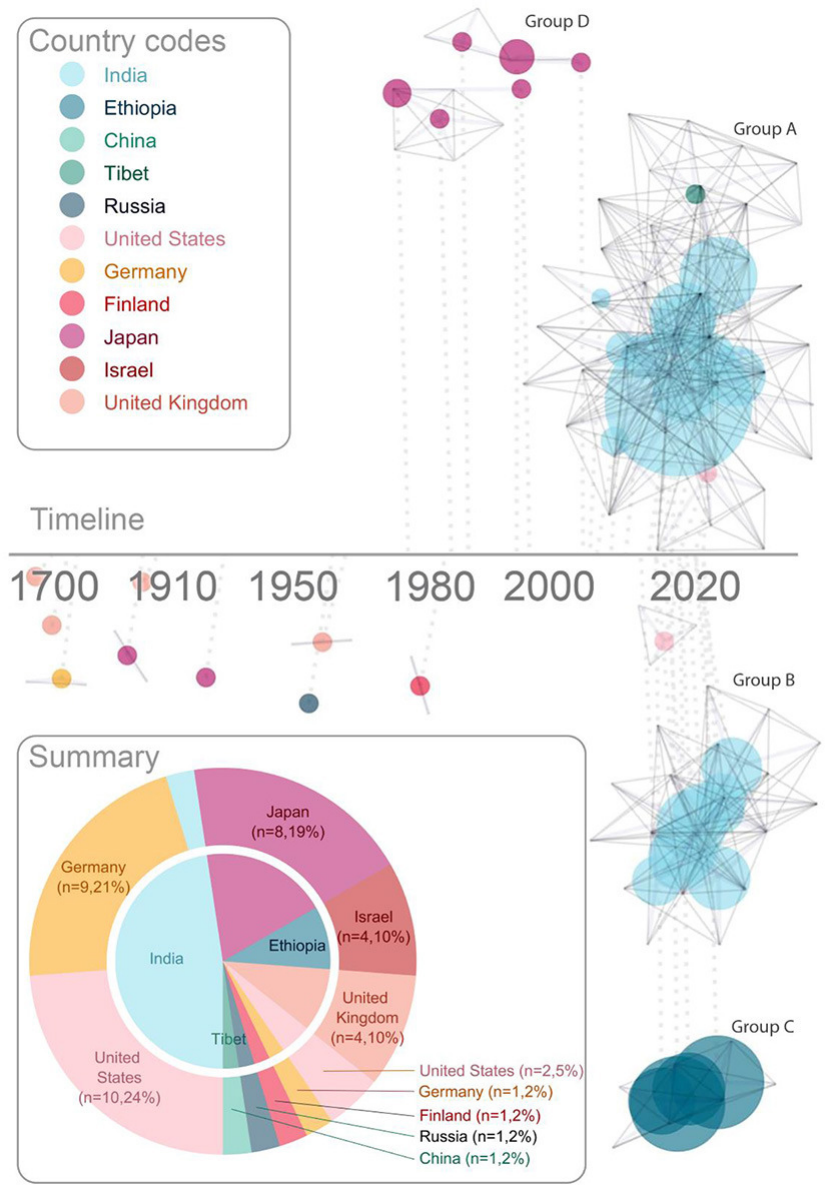
Characteristics of publications. The timeline shows each publication linked to the (logarithmic) timeline by a dotted line. Each vertex represents an author, and co-authorship is represented with solid gray lines. This network analysis highlights collaborative research teams. Each research team with 2 or more publications is assigned a letter [**(A–D)**, label color reflects country of institute with which the corresponding author affiliates]. Publications are represented with a dot, connected to each contributing author, the size of which represents the number of participants, and the color represents the country of the majority of the participants. The summary (lower left) shows number of publications by country; the outer ring shows country of institute with which the corresponding author is affiliated, and the inner pie shows the corresponding country of the majority of sight recovery participants for each study. Colors assigned to country are consistent across pie chart summary and network analysis timeline, as highlighted by country codes in the legend.

Visualizing the characteristics of the publications (Figure 2), reveals important context for this field. Given the longstanding philosophical interest in sight recovery, it is not surprising that there are several early publications following advances in cataract surgery. Published reports from Cheselden (1728), Wardrop (1826), Franz and August (1841), Motora and Matsumoto (1896), Latta (1904), Kuroda (1930), London (1960), Gregory and Wallace (1963), Carlson and Hyvarinen (1983) describe the challenges and joys of sight recovery through detailed case studies. These papers were generally written by treating surgeons (or close colleagues), and suggest a warm relationship, and co-location, between author and patient.

A shift toward more specialized research labs emerging for study of sight recovery started in Japan (Group D in Figure 2). Although not as highly cited as most papers in this field (Oyama et al., 2005), a small group of psychology researchers in Japan detailed the development of vision after sight recovery across multiple publications from the 1970's to the early 2000's (Mochizuki, 1976, 1997; Sasaki, 1984; Umezu, 1987; Torii and Mochizuki, 1995; Mochizuki and Torii, 2005). The shift from clinical case study to specialized academic research teams took full effect in 2006 (Ostrovsky et al., 2006) with an early publication from Project Prakash (Group A in Figure 2). This marked the beginning of large research groups (typically affiliated with universities in high-income countries) focusing on increasingly more specific visual skills, measured in a larger groups of participants (typically in lower-income countries).

##### Characteristics of participants

**Figure 3:**
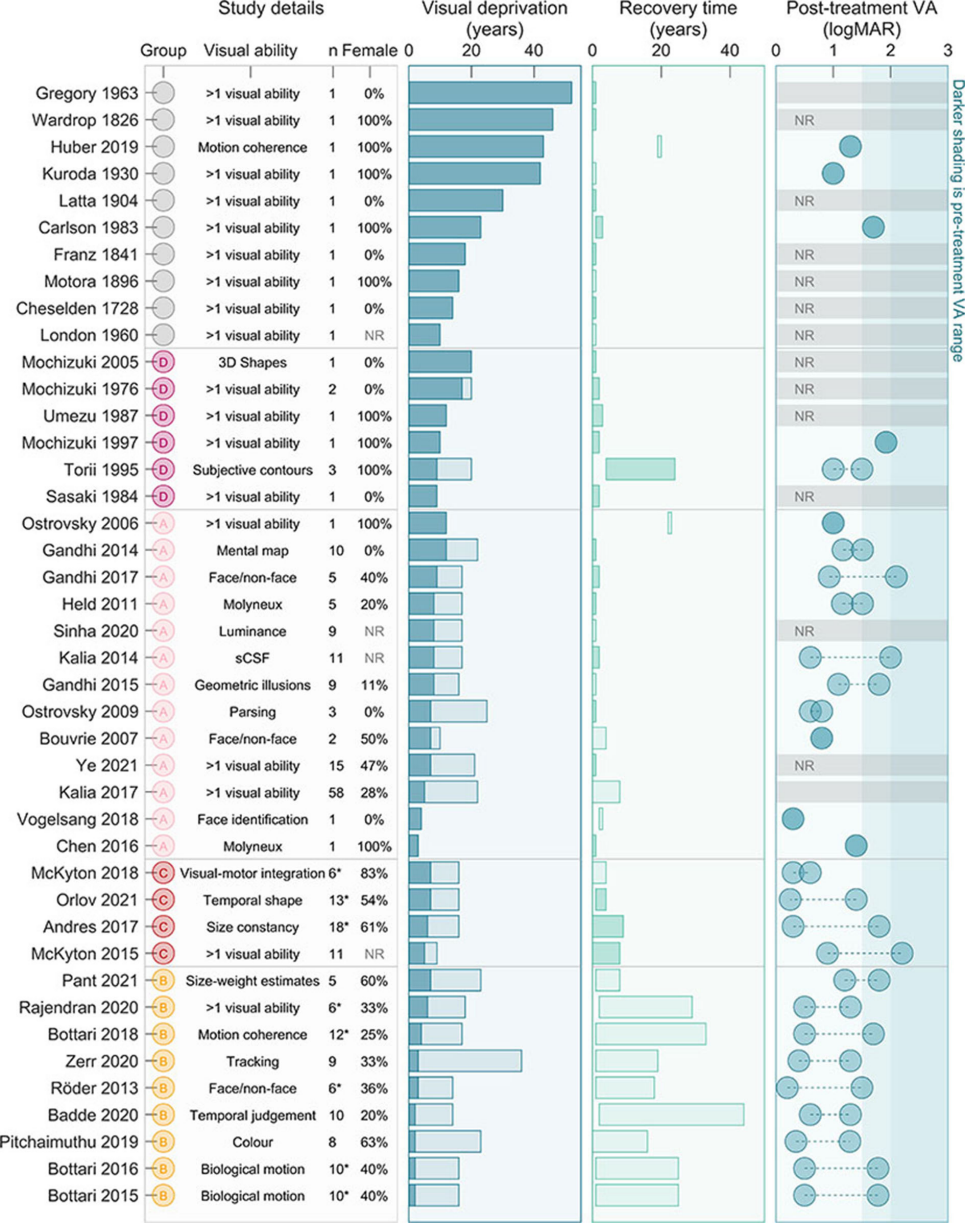
Characteristics of participants. In the **(left-most)** column “study details” we highlight the research group (based on those highlighted in Figure 2), the visual ability focused on, as well as the number of participants and percent female. In the second column “visual deprivation,” we include the range for each study of duration of visual deprivation, from 2 years (inclusion criteria) or minimum from study to the maximum for study. The third column “recovery time” displays time between ocular treatment and participation in the research project. Darker shaded bars indicate the study noted changes over time. The fourth **(right-most)** column “Post-treatment VA” shows the range of estimated VA outcomes for participants (in some cases notation was inferred before conversion, so logMAR values are estimates only). The darker shaded region is the estimated pre-surgical acuity across studies. NR, Not reported; VA, Visual Acuity; ^*^only a subset of participants met our criteria, but analysis (excluding duration, recovery time, and VA presented here and in Figure 4) included all participants.

Figure 3 summarizes participant characteristics by study. Studies ranged from describing a single case (Cheselden, 1728; Wardrop, 1826; Franz and August, 1841; Motora and Matsumoto, 1896; Latta, 1904; Kuroda, 1930; London, 1960; Gregory and Wallace, 1963; Carlson and Hyvarinen, 1983; Sasaki, 1984; Umezu, 1987; Mochizuki, 1997; Mochizuki and Torii, 2005; Ostrovsky et al., 2009; Chen et al., 2016; Vogelsang et al., 2018; Huber et al., 2019) to a large study including 58 participants, (Kalia et al., 2017) with a median of four participants. Ninety-one percent of included studies reported participant gender. Of those, a total of 241 persons participated, 91 of whom were female (38%). The duration of visual deprivation ranged from two (inclusion criteria) to 52 years (Gregory and Wallace, 1963). The majority of studies reported visual abilities within the first year of ocular treatment, while others measured visual ability as long at 44 years after (Badde et al., 2020).

Reported VA before ocular treatment was often only estimated, and generally ranged from counting fingers to light perception (LP). Some studies (particularly from Group C) reported better pre-treatment VA, however we suspect this reflects methodological rather than perceptual differences. Thirty studies reported VA after ocular treatment (71%). When reported, VA ranged from 0.2 logMAR (Röder et al., 2013) to LP (Carlson and Hyvarinen, 1983). A couple of studies included partipants with residual ocular pathology likely to limit VA (Carlson and Hyvarinen, 1983; Huber et al., 2019), however most publications since 2006 specifically excluded potential particpants with reisdual ocular, systemic or cognitive issues limiting visual function (excluding nystagmus and alignment issues, commonly associated with congenital blindness).

**Figure 4:**
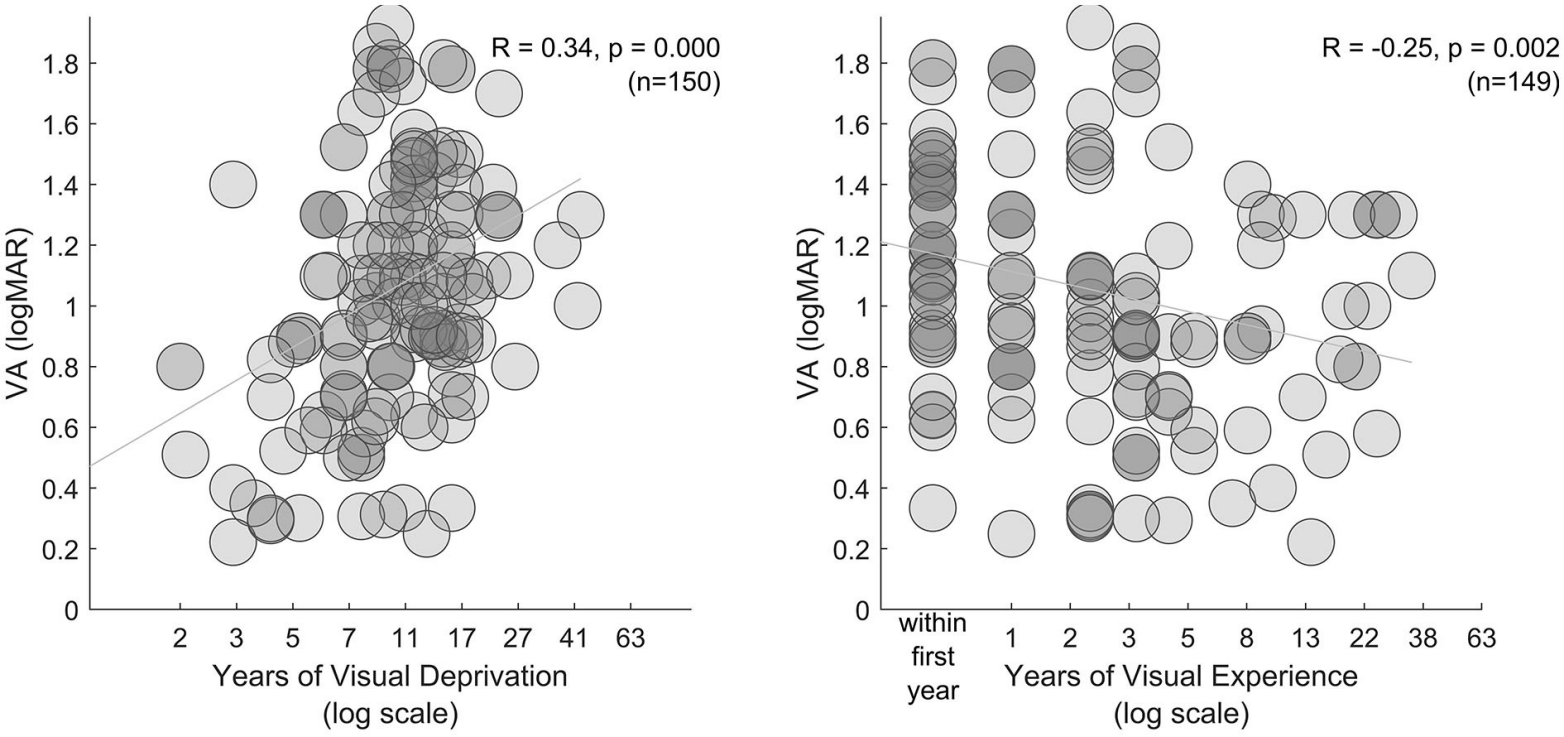
Extent of visual deprivation. In the left and middle subplots, VA is plotted against years of visual deprivation and years of visual experience (between ocular treatment and assessment), respectively.

Limiting participant level data to individuals with pre-treatment VA poorer than 1.5 logMAR (or unstated), as well as reported post-treatment VA and duration of deprivation and expereince, we narrowed our meta-analysis to 149 participants. Mean post-treatment VA in this group was 1.06 logMAR (±0.41 logMAR *StD*). As expected, longer duration of deprivation was associated with poorer VA (Figure 4, left, R = 0.34, *p* < 0.001). Overall, post-treatment visual experience was associated with better VA (Figure 4, right, R = −0.25, *p* = 0.002), however, this result could be driven by participants having poor acuity closely following ocular treatment. Excluding these participants, mean VA was slightly better (1.00 logMAR, ±0.42 logMAR *StD*), duration of deprivation was associated with poorer VA (R = 0.36, *p* < 0.001), and years of subsequent experience was not associated with improved VA (R = −0.16, *p* = 0.100). Within a multiple linear regression model only duration of deprivation significantly accounted for variation in VA (Figure 4, right). Note that participant data from research groups tended to cluster together, suggesting some individuals participanted in more than one study.

#### Question 2: How are visual abilities measured and categorized?

All but one study (Kalia et al., 2017–which used a questionnaire) measured a behavioral response to a visual stimulus. Several studies used physiological measures in combination with behavioral results (Röder et al., 2013; Bottari et al., 2015, 2016, 2018; Huber et al., 2019). These explored fMRI data on motion sensitive area hMT+ (Huber et al., 2019), and electrophysiological response to faces (Röder et al., 2013), biological motion (Bottari et al., 2015, 2016), and motion coherence (Bottari et al., 2018).

Case studies without comparators (Cheselden, 1728; Wardrop, 1826; Franz and August, 1841; Motora and Matsumoto, 1896; Latta, 1904; London, 1960; Gregory and Wallace, 1963; Mochizuki, 1977; Carlson and Hyvarinen, 1983; Umezu, 1987; Mochizuki and Torii, 2005), and to some degree more recent neuroscientific description of single cases (Chen et al., 2016; Vogelsang et al., 2018) focused on functional abilities, or the impact on sight recovery participants. By contrast, studies with a comparator tended to have a more anatomical focus, referring to hierarchical (low, mid or higher-level visual processing) and multisensory integration (with a focus integration of visual with auditory and motor development) framing.

Our attempt to simplify the visual abilities discussed within included studies, and place them within a parallel processing framework was challenging. At the streams level, because very few studies framed visual skills within a parallel streams framework, judgements were made by our author team, from a functional vision (rather than anatomical) perspective, based on existing literature (Grossmann, 2021; Pitcher, 2021; Pitcher and Ungerleider, 2021; Weiner and Gomez, 2021). At the visual skill level, tasks used to measure performance varied widely, with more use of custom than standardized assessment tools. Measurement of color perception, for example, ranged from asking a patient to name the color of died silk (Franz and August, 1841), to a custom psychophysical hue discrimination threshold task (McKyton et al., 2015) to use of a standardized Farnsworth 15 Color Vision test (Pitchaimuthu et al., 2019).

**Figure 5:**
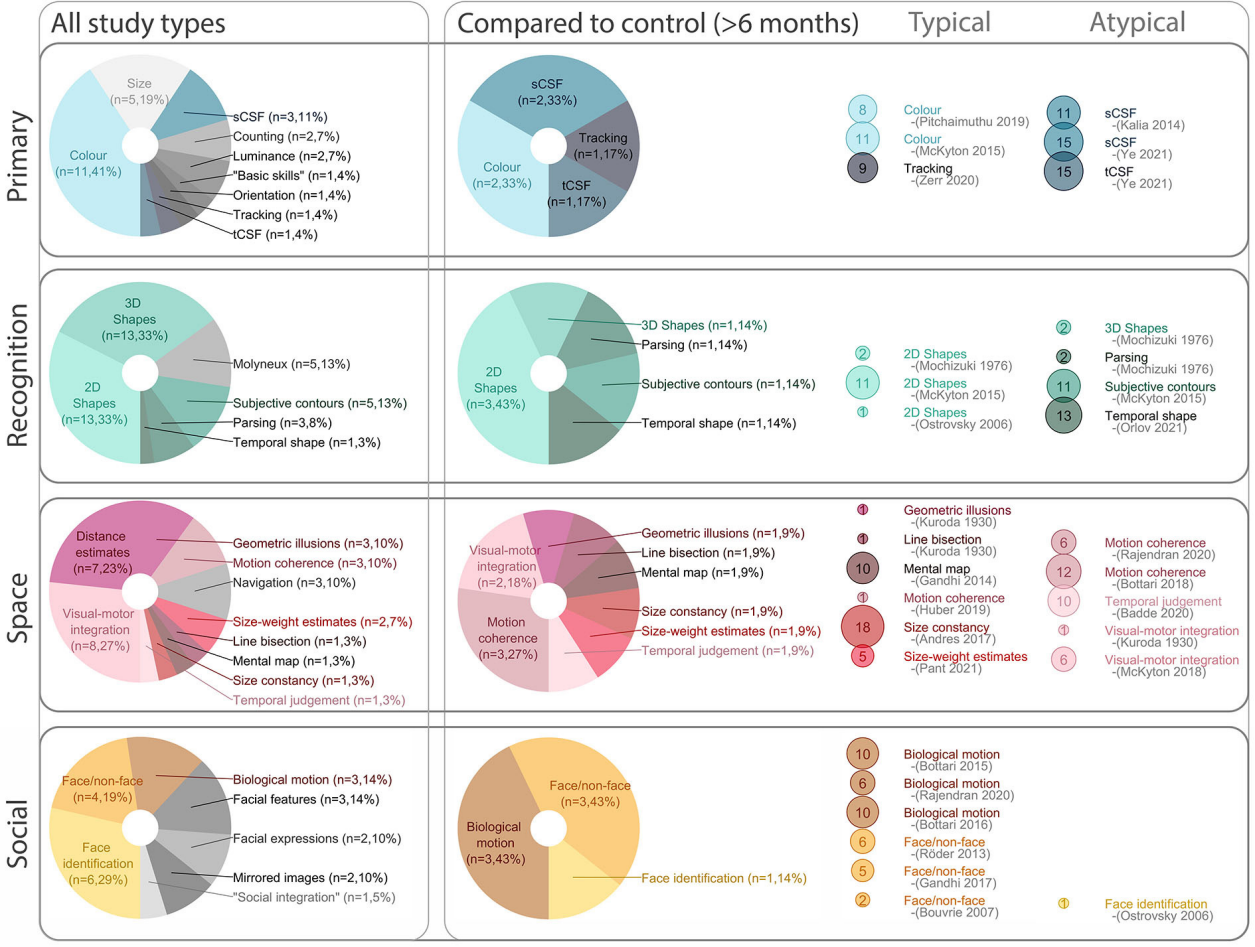
Visual abilities overview. The **(left)** column breaks down visual skills discussed across the review. The **(middle)** column zeros in on the subset of visual abilities which were compared to a control group (after the first 6 months of sight recovery). Of the visual skills compared to controls, the right column categorizes them as “Typical” (reported within variability of control group) or “Atypical” (reported outside variability of control group) for each specific task. In the **(right)** panel, dot size represents the number of participants (meeting our inclusion criteria) for each visual skill assessed. sCSF, spatial contrast sensitivity function; tCSF, temporal contrast sensitive function.

Shortfalls of classification notwithstanding, summarized data is presented in Figure 5-left. Across all included studies, we found that it was common to discuss some aspect of object recognition, which we grouped as 2D (simple shapes), and 3D (2D representations of 3D objects, or real objects). Performance of sight recovery participants was specifically compared to a reference group for over half of the visual skills discussed. For this subset, assessed visual skills were more diverse; we found a relatively equal exploration of:

1. “Primary” [including spatial (Kalia et al., 2014; Ye et al., 2021) and temporal (Ye et al., 2021) contrast sensitivity, color perception (McKyton et al., 2015; Pitchaimuthu et al., 2019) and eye movements (Zerr et al., 2020)];
2. “Recognition” [including understanding of shapes and objects, both 2D (Mochizuki, 1976; Ostrovsky et al., 2006; McKyton et al., 2015) and 3D, (Mochizuki, 1976) with various degrading influences including subjective contours (Mochizuki, 1976; Torii and Mochizuki, 1995) as well as temporal shape recognition (Orlov et al., 2021)];
3. Understanding of visual “space” [including motion coherence thresholds, (Bottari et al., 2018; Huber et al., 2019; Rajendran et al., 2020) temporal judgements, (Badde et al., 2020) visual-motor integration, (Kuroda, 1930; McKyton et al., 2018) size constancy, (Andres et al., 2017) susceptibility to geometric (Kuroda, 1930) and size-weight (Pant et al., 2021) illusions, and the ability to establish and use information stored as mental maps (Gandhi et al., 2015)]; and,
4. “Social” aspects of vision [including biological motion, (Bottari et al., 2015, 2018; Rajendran et al., 2020) face/non-face discriminations; (Bouvrie and Sinha, 2007; Röder et al., 2013; Gandhi et al., 2017) and, face identification (Ostrovsky et al., 2006)].

#### Question 3: Which visual abilities are likely to be compromised by (and which are likely to be resilient to) early and extended bilateral visual deprivation?

Several studies demonstrated dramatic improvements in visual abilities before to after ocular treatment (Gandhi et al., 2014, 2017; Andres et al., 2017; Kalia et al., 2017; Ye et al., 2021), as well as further improvements within the first few months of recovery (Cheselden, 1728; Sasaki, 1984; Ostrovsky et al., 2009; Gandhi et al., 2014, 2015, 2017; Kalia et al., 2014; McKyton et al., 2015, 2018; Chen et al., 2016; Andres et al., 2017; Sinha et al., 2020; Ye et al., 2021) and ensuing years (Mochizuki, 1976, 1977, 1979, 1997; Sasaki, 1984; Sasaki et al., 1994; Torii and Mochizuki, 1995; Mochizuki and Torii, 2005). When we focused only on the subset of visual abilities which were compared to controls, and representative of outcomes at least 6 months after ocular treatment, a profile of more enduring areas of resilience and challenge emerges.

In terms of resilient visual abilities after initial treatment, tracking and saccadic eye movements after nystagmus were accounted for (Zerr et al., 2020), comparative judgements about luminance and color (McKyton et al., 2015; Pitchaimuthu et al., 2019; Sinha et al., 2020) and recognition of simple 2D shapes (Mochizuki, 1976; Ostrovsky et al., 2006; McKyton et al., 2015) tended to be within the range of typically developing controls. Similarly, biological motion was reported as remarkably intact [inducing detection (Bottari et al., 2015, 2016) and interpretation of specific actions (Rajendran et al., 2020)] as was the ability to differentiate between faces and non-faces (Bouvrie and Sinha, 2007; Röder et al., 2013; Gandhi et al., 2017). Sight recovery participants appeared susceptible (potentially more susceptible) to classic geometric illusions [Ponzo and Müller-Lyer illusions (Gandhi et al., 2015) and the size-weight estimates (Pant et al., 2021)], and performed well on line bisection tasks (Kuroda, 1930) and using mental maps to recall spatial relationships (Gandhi et al., 2014).

A variety of visual skills were reported as challenging. There are well-documented deficits in spatial contrast sensitivity (Kalia et al., 2014; Ye et al., 2021) (reduced at low and high spatial frequencies, and therefore impacting cut-off frequencies, in line with reduced VA). To a lesser extent, temporal contrast sensitivity is also appears different from controls (cut-off frequencies appear intact, but detection of low temporal frequencies remains compromised, Ye et al., 2021). Complex recognition tasks (including 3D and obscured shapes (McKyton et al., 2015), temporally defined shapes (Orlov et al., 2021) and potentially face identification (Ostrovsky et al., 2006) appear a particular challenge after sight recovery, although there is some evidence of improvement after years to decades of visual experience (Ostrovsky et al., 2006; Orlov et al., 2021). Performance on motion coherence tasks were generally found to be poor (Bottari et al., 2018; Rajendran et al., 2020). However, a single participant was reported to achieve a result in the typically developing range 20 years after sight recovery (Huber et al., 2019). Some aspects of multi-modal integration (judgements of the relative timing of visual vs. auditory and tactile signals (Badde et al., 2020), and visual motor integration (Kuroda, 1930; McKyton et al., 2018) are also compromised.

**Figure 6:**
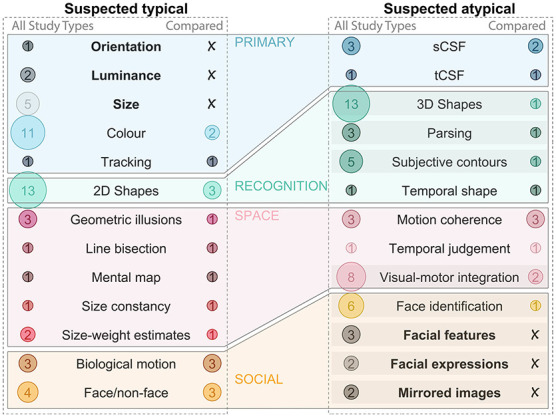
Expected outcome summary. The **(left)** column includes all visual skills suspected to be typical after visual deprivation. The bubbles on the left of each subplot highlight the number of studies which discussed this skill across all study types, the bubbles on the right of each subplot highlight the number of studies which compared this skill to a control group. Visual skills which have been discussed in the literature, but not compared to a control group are bolded, and those which are suspected to be atypical and are likely in need of more research are highlighted with a shaded bar. Only visual skills which remain relevant more than 6 months after the ocular treatment are included (e.g., Molyneux's question is excluded). sCSF, spatial contrast sensitivity function; tCSF, temporal contrast sensitive function.

Although the method of observation ranged widely, and reported results were not always consistent for each visual skill, some wider trends emerged (Figure 6). Overall, perception of illusions, orientation, size (or length), contours (2D shapes) and color is generally reported as typical, whereas interpretation of depth cues (3D shapes), parsing superimposed images and understanding nuance in faces is more challenging, suggesting that surface-based (rather than image-based) processing is impacted by extended visual deprivation. Among visual skills expected to be atypical, some have been explored with more rigor than others. For example, many studies noted that 3D images were challenging, while few compared performance to a control group. Similarly, image segmentation/parsing, and various aspects of face processing beyond face/non-face discrimination are often noted as challenging in case studies, but are less of a focus in controlled studies (highlighted in Figure 6).

#### Question 4: Through what disciplinary lens was the research undertaken and interpreted?

Most studies discussed several potential implications of their work. Those that referenced neurodevelopmental insights (*n* = 31) were more closely connected to those that discussed the impact of timing of ocular treatment on visual skills (*n* = 15). Those that referenced (re)habilitation (*n* = 28) tended to also discuss quality of life (*n* = 11). Neurodevelopmental insights and quality of life were least connected. Highlighting a key focus for each paper, we found more than half were primarily interested in neurodevelopmental insights (*n* = 26), and only a handful focused on quality of life (*n* = 5). If we consider study characteristics (Question 1), type of study (Question 2) and key discussion points (Question 4) together, there appear to be three clusters of work, described below.

Studies seeking neurodevelopmental insights tended to use quantitative methods with control groups [except those interested in philosophy (Held et al., 2011) or modeling (Vogelsang et al., 2018)], and focus on a specific visual skill (framed in terms of hierarchical processes or multisensory integration). These studies tended to be more recent, include larger groups of participants, and the corresponding author tended not to be affiliated with a university located in the same country as the participants. Generally, these studies aimed to *reveal a more general truth* about how the brain responds to visual deprivation, perhaps aligned with “positivism” (Alharahsheh and Pius, 2020).

By contrast, studies focused on patient experience, tended to use qualitative approaches to describe longitudinal outcomes, across a wide range of functional visual skills, and what this meant for the participants in terms of their quality of life. These publications were generally older, with fewer participants, most of whom lived in the same country as the corresponding author affiliation. These studies were generally describing *personal experience* after sight recovery, perhaps aligned with an “interpretivism” lens (Alharahsheh and Pius, 2020).

Studies from Japan bridge this gap both in terms of historical context and disciplinary lens. These studies typically conducted a battery of functional tests including spatial perception (Motora and Matsumoto, 1896; Kuroda, 1930; Sasaki, 1984; Sasaki et al., 1994), visual illusions (Motora and Matsumoto, 1896; Kuroda, 1930), shape recognition (Motora and Matsumoto, 1896; Mochizuki, 1976, 1979, 1997; Sasaki, 1984; Umezu, 1987; Sasaki et al., 1994; Torii and Mochizuki, 1995; Mochizuki and Torii, 2005), color perception (Motora and Matsumoto, 1896; Kuroda, 1930; Sasaki, 1984; Umezu, 1987; Sasaki et al., 1994) face perception (Motora and Matsumoto, 1896; Kuroda, 1930), and recognition of mirror images (Mochizuki, 1997), from more of a positivism framework, but generally without control groups. These studies used a longitudinal approach, and had more of a focus on (re)habilitation than more recent work.

##### Quality of life, (re)habilitation and attitudes toward sight recovery

Early work describing the experience of sight recovery painted a fairly melancholy picture; Cheselden (1728), Wardrop (1826), Latta (1904), Gregory and Wallace (1963), and Carlson and Hyvarinen (1983) did not shy away from describing the profound challenges faced after ocular treatment. Several referenced patients experiencing increased anxiety (in some cases profound depression, Gregory and Wallace, 1963) speculated to be related to the disconnect between expectations (of others and themselves) and their lived experience, and a grappling with how the new information contradicted fundamental beliefs about the physical environment. Duration of deprivation likely contributes to the psychological challenges; Cheseldon's description of a teen suggested better functional outcomes than Gregory's description of a man in his fifties. A more recent study was considerably more optimistic; Kalia et al. (2017) explored the personal impact or sight recovery by asking participants to estimate how their lives changed after ocular treatment. Almost all participants reported improvements, and the authors argue that providing surgery at any point in life (participants in this study had experienced between 5 and 22 years of visual deprivation) improves quality of life (Kalia et al., 2017).

Studies focused on patient experience generally advocated for supportive networks and active (re)habilitation for successful re-engagement with educational and social systems. Much of the focus on (re)habilitation was on various aspects of recognition and visual motor integration (Sasaki, 1984; Sasaki et al., 1994; Orlov et al., 2021). Several studies noted importance of touching objects (Wardrop, 1826; Held et al., 2011; Chen et al., 2016), physically tracing outlines of objects in the air (Latta, 1904) or on paper (Carlson and Hyvarinen, 1983), moving objects around (Carlson and Hyvarinen, 1983; Ostrovsky et al., 2009) and exploring them from different perspectives (Torii and Mochizuki, 1995; Mochizuki and Torii, 2005) to facilitate the visual learning of object identification, binding and parsing. Some discussion in the text focused on the attitudes of participants about sight recovery (Cheselden, 1728; Franz and August, 1841; Latta, 1904; Carlson and Hyvarinen, 1983). For example, Latta (1904) contrasts the experience of two siblings who responded very differently to sight recovery; he speculated that pre-treatment immersion with sighted peers, and curiosity about the visual world facilitated successful (re)habilitation.

##### Neurodevelopmental impact of sight recovery

Although some more theoretical studies were interested in what sight recovery could tell us about perceptual phenomena [e.g., whether geometric illusions were likely to be “top-down” vs. “bottom-up” (Gandhi et al., 2015; Sinha et al., 2020)], most were interested in the role of visual experience in the development of the human visual system. Researchers tended to pose the historic idea of a “pure' critical period (that early visual experience is required for refined visual development, based largely on early animal models), then hypothesize what underpinned exceptions.

The most basic explanation was that residual pre-treatment vision is sufficient for measured performance. The light that passes through even very dense cataracts was noted to have a potentially facilitating effect for judgements of color (Pitchaimuthu et al., 2019), basic size estimates (Andres et al., 2017), and perception of high temporal frequency (Ye et al., 2021). Evolutionary context was also raised; understanding complex objects (Ostrovsky et al., 2009; Orlov et al., 2021) and faces (Röder et al., 2013) (skills reportedly challenging after a period of visual deprivation) are context specific, and therefore best learned by experience, whereas the ability to understanding biological motion (a reportedly resilient skill) may evolutionarily be context agnostic, and develop independently of visual experience (Bottari et al., 2015; Rajendran et al., 2020). Several studies noted that interactions between senses could facilitate high-level visual abilities, perhaps connected to Rauschecker and Harris's (1983) finding of faciliatory, rather than competitive, intermodal interaction between auditory and visual inputs in the superior colliculus of cats. New visual input may successfully map to existing neural architecture for motor function (potentially facilitating visual perception of biological motion, Bottari et al., 2015; Rajendran et al., 2020) and directionality of moving sound (potentially facilitating visual perception of motion coherence, Bottari et al., 2018; Huber et al., 2019; Rajendran et al., 2020). Several studies commented on whether retained visual abilities had similar underlying mechanisms to that in typically developing peers. Studies including electrophysiological analysis suggested that face perception (Bouvrie and Sinha, 2007; Röder et al., 2013) and motion coherence had different underlying mechanisms between groups (suggesting compensatory strategies), whereas biological motion appeared physiologically indistinguishable (Bottari et al., 2015, 2016).

## Discussion

We wanted to understand how published literature about sight recovery could inform updates in low vision (re)habilitation programs for children after delayed access to ocular treatment. Anticipating insufficient evidence to determine effectiveness of particular strategies, we undertook a scoping review of studies including participants who have experienced early and extended visual deprivation to understand existing information.

### Summary of evidence

The meta-analysis of patient level data (Question 1) provided insight into expected VA. Post-treatment VA depended on duration of visual deprivation more than years of port-treatment visual experience, with average VA around 1 logMAR. Categorizing extra striate visual skills assessed in each study (Question 2) and summarizing reported outcomes for each (Question 3) provided insight into expected visual processing challenges. There is evidence for persistent deficits in contrast sensitivity, integration of motion cues, and visual-motor integration, with additional suspected deficits in interpreting complex 3D shapes, parsing, subjective contours and understanding identity and expression of faces. There is evidence for resilience in color perception, understanding of simple shapes, discriminating between a face and non-face, and the perception of biological motion. Basic measures of orientation, luminance, size, are also suspected to be intact after early and extended bilateral visual deprivation. Mapping insights by disciplinary lens (Question 4) added important context. From an experiential perspective, there is evidence that ocular treatment after early and extended bilateral visual deprivation improves quality of life, but the literature also makes it clear that some people struggle with the transition and need support. Active (re)habilitation strategies mentioned were persistent practice and multi-modal training, but neither were evaluated. Insights from the neurodevelopmental perspective provide reason for optimism; many studies pointed to a capacity to develop visual skills well-beyond what was traditionally thought of as the “critical period.”

### Future research

The above evidence establishes a starting point for discussing appropriate expectations with families, and developing clinical/low vision assessment tools and targeted (re)habilitation strategies. Considering our findings in the context of wider literature, we propose several avenues for future research, summarized within three categories.

#### Equitable access to ocular treatment

Continued research, service and policy initiatives to improve access to childhood ocular treatment are needed to minimize the impact of bilateral visual deprivation. Consistent with previous work (Lewis and Maurer, 2005, 2009; Maurer et al., 2005, 2007; Maurer, 2017; Allen, 2020), we highlight the impact of duration of visual deprivation on VA, and persistent deficits in some extra-striate visual abilities after extended visual deprivation. Prompt access to pediatric ophthalmologic services will improve outcomes. Further, the gender disparity in our metanalysis is consistent with a known trend that boys are brought for cataract surgery at a higher rate than girls (Gilbert and Lepvrier-Chomette, 2016). Reporting on gender, and implementing interventions to promote prompt surgical services for girls (Reddy et al., 2018) are particularly important given the amplifying impact of delays on visual outcomes.

#### Understanding extra-striate visual abilities in a (re)habilitation context

Continued research about the impact of visual deprivation on specific visual skills will help to zero in on relevant visual abilities to assess in this population. This could initially be focused on visual abilities that pose a challenge for patients in early case studies, but which have not been followed up on in controlled studies, such as interpretation of complex objects and faces. Related literature suggests particular challenges in interpreting social cues (Geldart et al., 2002; Le Grand et al., 2004; Huber et al., 2015).

Linking isolated visual abilities to integrated functional outcomes would help to further prioritize which visual abilities should be targeted for counseling, assessment and (re)habilitation. Although we have summarized a wide range of visual skills in this review, some are more relevant for daily function and quality of life than others (Merabet and Pascual-Leone, 2010). Considering how these visual skills translate to day to day tasks, understanding patient goals, and matching patient expectations with probable functional outcomes, are all important for a patient to experience the ocular treatment as a success.

The development of assessment tools that are appropriate for impacted patients, and practical for community context is an important step in translating findings. Part of the challenge of interpreting data from this field is the diversity of assessment tools used. There is a gap between custom psychophysical tasks designed to measure threshold performance on a single visual skill (common within positivism approaches), and the more interactive battery of less controlled assessments designed to gain insight into a wide range of functional abilities (used in the more interpretivism research). Some attempts to bridge this gap exist (e.g., CVIT 3–6, Vancleef et al., 2020). Adoption of existing tools will likely require modifications for culture (Duke et al., 2021) and low-level abilities to be appropriate for impacted children.

#### (Re)habilitation

More focus on (re)habilitation is needed. Newer research is increasingly done by academics affiliated with institutes in high-income countries, while participants are mainly in low-income countries. This makes sense–there is less avoidable childhood blindness in high-income countries, and collaborations help fund ocular treatment for people with limited access. However, this trend carries some risks–some of which are highlighted by the founder of the largest of these research groups (Sinha and Held, 2012; Sinha et al., 2013).

“*A project that merges science and service risks being misinterpreted as exploitative or disrespectful of cultural and religious traditions, especially in developing countries where there is greater resistance to science based interventions due to competing religious, cultural, or community beliefs. This very real danger must be countered with clear communication between scientists and society. In Project Prakash, we find that counselling parents, a frank description of the project's objectives, and a few local non-scientist champions whom the patient population can relate to, are often necessary and sufficient to address this concern.”* –(Sinha et al., 2013).

In addition to risks described by Sinha et al. (2013), we noted that the distance between research and participant co-occurred with a change in disciplinary approach, with a shift away from research about quality of life and (re)habilitation, toward more neuroscientific insights relevant beyond the participants themselves. Both approaches are necessary, but we argue there is currently an opportunity to translate recent insights into patient-centered implementation research to improve services directly for impacted children. Website scans confirm some of the research groups highlighted in this review are working on (re)habilitation strategies, so we are optimistic more research is coming. This review suggest learning to see can be challenging. Wider research suggests creative solutions (e.g., gamification, integrating senses, tangible measures of progression) will need to be explored to optimize (re)habilitation efforts (Jeon et al., 2012; Beyeler et al., 2017; Ciman et al., 2018; Hamm et al., 2018).

Continued neuroscientific research about the mechanisms of visual development and plasticity are needed to facilitate the quality low vision services into the future. Although the bounds of plasticity provide continued optomism (Beyeler et al., 2017), constraints still exist and it appears practice alone may be insufficient to improve certain visual skills (Šikl et al., 2013). Although not currently actionable in a (re)habilitation setting, there is growing evidence that plasticity within the human visual cortex can be enhanced by manipulating biochemical constraints (Castaldi et al., 2020). It will be exciting to follow these advances to find novel ways to facilitate sight recovery after early and extended bilateral visual deprivation in the future (Castaldi et al., 2020; Heimler and Amedi, 2020), continuing to enable basic science to inform (re)habilitation practice (Rauschecker, 1999; Merabet and Pascual-Leone, 2010).

### Study limitations

Our search strategy was simple, however our extensive searching of referenced and citing publications of included studies mitigated potential risk of bias.

Our inclusion criteria limited the type of information synthesized in several ways. We excluded studies in which childhood blindness began after the age of 1 year (e.g., Valvo, 1968; Fine et al., 2003), was short in duration (e.g., de Heering et al., 2016; Hadad et al., 2017), or was the result of sight-substitution devices (e.g., Reich and Amedi, 2015). We did not include abstracts, gray literature, or books (e.g., Von Senden, 1932; Valvo, 1971; Lester, 1972; Sacks, 1995), or the substantial clinical work about outcomes after cataract surgery (e.g., Gogate et al., 2014). These inputs would provide different, and potentially useful, perspectives. Specifically, some of these resources speak to the psychological impact of visual restoration (Lester, 1972) and potential (re)habilitation strategies (Reich and Amedi, 2015). We reasoned that narrow inclusion was important, as wide participant heterogeneity could dilute findings relevant to our target population. Despite our targeted approach, even within included studies confirmation of congenital onset remained somewhat speculative. This may change in the future, as objective measures associated with congenital onset of blindness are being developed (Sourav et al., 2020).

Finally, the simplifications used in this review (particularly in terms of visual skills and patient groups) downplay important nuance. As we translate insights from this field into (re)habilitation services, it will be important to use synthesized research to inform an individualized patient-centered approach.

## Conclusion

Early and extended visual deprivation impacts the development of visual processing, with persistent visual processing challenges long after sight has been restored. There is evidence for persistent deficits in contrast sensitivity, complex shape and motion coherence, compared to resilience within color perception, understanding 2D shapes, face/non-face discrimination, and biological motion perception. The literature summarizing this experience provides several insights to start to refine low vision services to best support the process of learning to see, but more research is needed. There is a current opportunity to start to translate the recent insights about neurodevelopment into (re)habilitative strategies to more directly support impacted patients. Implementation of resulting strategies has the potential to enhance the impact of current initiatives providing sight-saving treatments for congenital blindness in areas where access to eye care is limited.

## Data availability statement

The original contributions presented in the study are included in the article/supplementary material, further inquiries can be directed to the corresponding author.

## Author contributions

EK, PA, RG, MS, and LH collaboratively conceptualized the project. EM, EK, PA, GM, and LH completed screening and data extraction. EM, GM, MS, and LH worked on the meta-analysis. LH, EM, GM, and MS drafted sections of the manuscript. All authors contributed to revision, and approved the submitted version.

## Funding

LH is a Rutherford Postdoctoral Research Fellow, funded by the Royal Society Te Apārangi (19-UOA-028) and School of Medicine Foundation (M-FMH-MGUP).

## Conflict of interest

The authors declare that the research was conducted in the absence of any commercial or financial relationships that could be construed as a potential conflict of interest.

## Publisher's note

All claims expressed in this article are solely those of the authors and do not necessarily represent those of their affiliated organizations, or those of the publisher, the editors and the reviewers. Any product that may be evaluated in this article, or claim that may be made by its manufacturer, is not guaranteed or endorsed by the publisher.
